# Functional MgO–Lignin Hybrids and Their Application as Fillers for Polypropylene Composites

**DOI:** 10.3390/molecules25040864

**Published:** 2020-02-16

**Authors:** Aleksandra Grząbka-Zasadzińska, Łukasz Klapiszewski, Teofil Jesionowski, Sławomir Borysiak

**Affiliations:** Institute of Chemical Technology and Engineering, Faculty of Chemical Technology, Poznan University of Technology, Berdychowo 4, PL-60965 Poznan, Poland; teofil.jesionowski@put.poznan.pl (T.J.); slawomir.borysiak@put.poznan.pl (S.B.)

**Keywords:** biopolymers, inorganic-organic hybrid materials, polypropylene, polymer composites

## Abstract

Inorganic–organic hybrids are a group of materials that have recently become the subject of intense scientific research. They exhibit some of the specific properties of both highly durable inorganic materials (e.g., titanium dioxide, zinc) and organic products with divergent physicochemical traits (e.g., lignin, chitin). This combination results in improved physicochemical, thermal or mechanical properties. Hybrids with defined characteristics can be used as fillers for polymer composites. In this study, three types of filler with different MgO/lignin ratio were used as fillers for polypropylene (PP). The effectiveness of MgO-lignin binding was confirmed using Fourier transform infrared spectroscopy. The fillers were also tested in terms of thermal stability, dispersive-morphological properties as well as porous structure. Polymer composites containing 3 wt.% of each filler were subjected to wide angle X-ray diffraction tests, differential scanning calorimetry and microscopic studies to define their structure, morphology and thermal properties. Additionally, tensile tests of the composites were performed. It was established that the composition of the filler has a significant influence on the crystallization of polypropylene—either spherulites or transcrystalline layers were formed. The value of Young’s modulus and tensile strength remained unaffected by filler type. However, composites with hybrid fillers exhibited lower elongation at break than unfilled polypropylene.

## 1. Introduction

Biodegradable materials/biomaterials, hybrid materials or composites/biocomposites obtained from renewable sources (biomass) are extremely promising products that may replace synthetic polymers in the future [1]. Recently, inorganic–organic hybrid materials have attracted much interest and gained importance not only in science, but also in technological sectors [2,3,4]. For this reason, materials based on biopolymers, primarily lignin, are being intensively developed [5,6,7,8]. The selection of this biopolymer is mainly dictated by its structural diversity as well as ease of preparation, which results from the production associated with cellulose fibers [9]. In addition, lignin is the second most available substance on Earth after cellulose [8]. Moreover, this biopolymer is distinguished from other products by properties such as biodegradability, antioxidant and antibacterial activity [10,11], good chemical reactivity [12], affinity to inorganic oxides [13,14] or possible sorption of harmful compounds from the environment due to the diversity of functional groups in the lignin structure [15,16]. These properties allow the use of lignin in the preparation of functional hybrid materials with applications as fillers for polymer composites [14,17,18,19] or effective sorbents of environmentally harmful metal ions [15,16]. It is worth emphasizing that lignin is a renewable raw material, i.e., it corresponds well with global environmental trends. However, a significant disadvantage of lignin is its limited thermal stability. At temperatures above ~200 °C this biopolymer is gradually decomposed, which ultimately results in its degradation [14,17,18,19]. Therefore, it seems important to combine lignin with inorganic compounds, including highly thermally stable MgO [20,21]. This gives the opportunity to create new inorganic–organic hybrid materials for specific applications.

Hybrid materials possess specific characteristics, resulting from the combination of inorganic (e.g., titanium dioxide, silicon, zinc, aluminum) and organic (e.g., lignin, chitin) particles. This combination contributes to improved physicochemical, thermal or mechanical properties. The presence of various chemical compounds allows systems with strictly defined characteristics to be obtained. Currently, hybrid materials are eagerly used as fillers for polymer matrices [7,13,14]. However, they are a relatively new type of polymeric fillers and, therefore, still need further, detailed research. 

Production of composites with hybrid fillers based on metal oxides is important in terms of increasing the thermal stability. Another aspect is the determination of the nucleation activity of the hybrid filler in the polymer matrix. This work is a continuation of our research regarding composites with metal oxide-lignin fillers which have already revealed that the type of metal oxide significantly influences the formation of the structure of polypropylene (PP) and its nucleating ability. It is believed that phenomena occurring at the filler/composite interface as well as the formation of the supermolecular structure of polymers will provide a better insight for designing materials with the assumed properties.

## 2. Results and Discussion

### 2.1. Characteristics of Inorganic–Organic Hybrid Materials and Pristine Components

#### 2.1.1. Fourier Transform Infrared Spectroscopy (FTIR)

**Figure 1:**
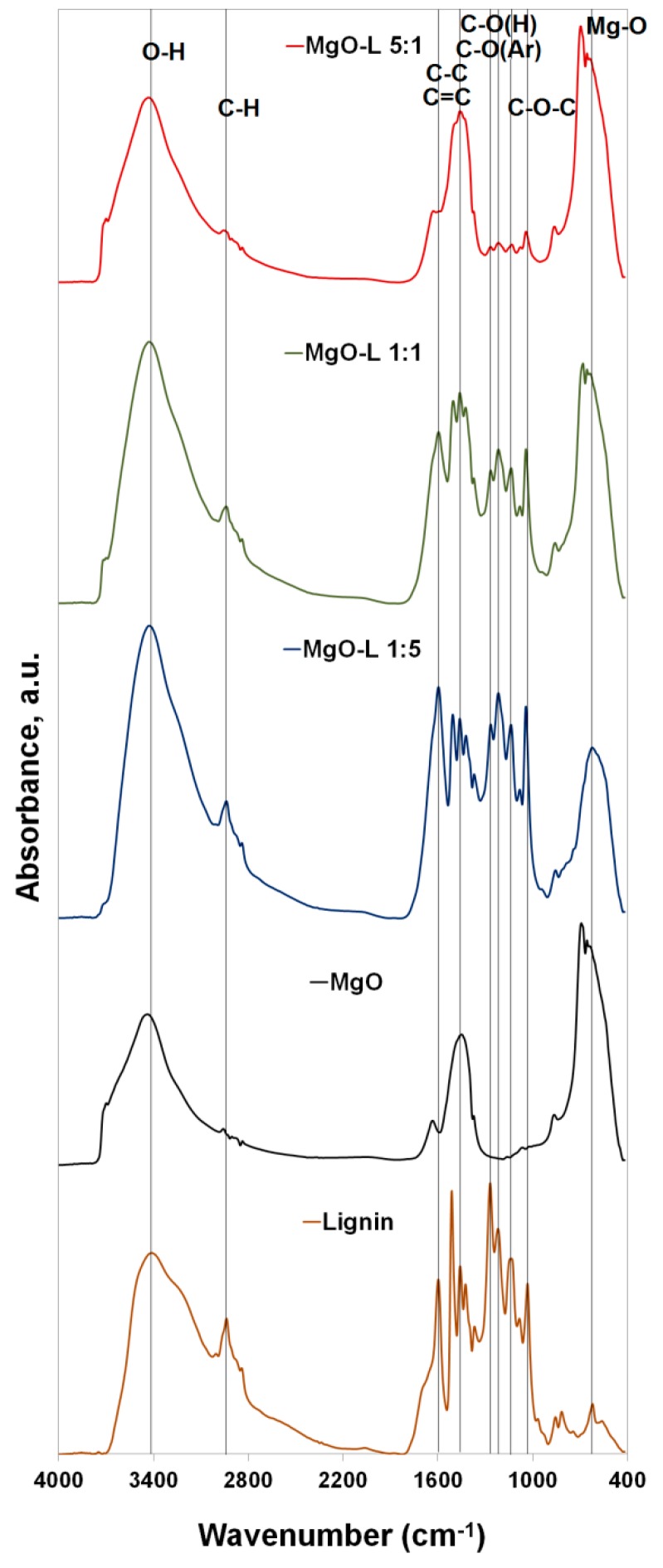
Fourier transform infrared (FTIR) spectra of MgO, lignin and MgO–lignin hybrid materials with different ratio of individual components.

Fourier transform infrared spectroscopy (FTIR) was used to identify the characteristic functional groups present in the structure of the MgO-lignin hybrid fillers produced as well as in pristine components i.e., magnesium oxide and lignin (see Figure 1).

In the case of all hybrid systems, characteristic signals originating from functional groups present in lignin were observed: stretching vibrations of O–H bonds in the wavenumbers range of 3600–3200 cm^−1^, stretching vibrations of C–H bonds, including those derived from -CH_3_, -CH_2_ and O–CH_3_ groups, with wavenumber values of 2950–2840 cm^−1^, vibrations associated with the presence of aromatic rings in the biopolymer structure, in the wavenumber range of 1600–1500 cm^−1^ and 1490–1440 cm^−1^. Among the recorded signals, bands derived from vibrations of various types of carbon atom bonding, including ether groups (C–O–C) in the range of 1080–1020 cm^−1^ and bands assigned to Ar–C–H bending vibrations (875–840 cm^−1^) also play an important role. A crucial conclusion which can be established based on the analysis of spectra for hybrid systems is that the intensity of the bands of groups characteristic for lignin decreases with the decreasing ratio of the biopolymer in relation to the inorganic matrix. This has already been observed in our earlier reports in which SiO_2_–lignin [11,13,15,17,18,19] or ZnO–lignin [14] hybrid materials were used. In each case, there was a permanent connection between the components, as a result of which hybrid materials belonging to the first class (with physical connection of components, mainly through hydrogen bonds) [14,17,18,19] or the second class (chemical combination of components with the formation of covalent bonds) were obtained, depending on the method used [13,15]. In the chemical method, it was important to modify the inorganic matrix properly and activate the biopolymer, which was mainly obtained by oxidation with inorganic compounds [13,15] or, alternatively, with ionic liquids [20]. In the case of the MgO–lignin systems used in this publication and their use as environmentally friendly polymer fillers, it is sufficient to physically connect the components without the need to conduct prior chemical modifications. This is confirmed by visible shifts in the absorption maxima of individual bands of characteristic groups, mainly hydroxyl (~3400 cm^−1^) and Mg–O (~670 cm^−1^) bonds (corresponding changes marked in Figure 1).

The presence of the band originating from O–H bonds, associated with the physically bound water at the oxide surface, was also observed in case of pure MgO (maximum intensity at wavenumber 3425 cm^−1^). Additionally, the band at ~1608 cm^−1^ also confirms the presence of physically adsorbed water on the MgO surface. The band at ~670 cm^−1^, which confirms the presence of bonds derived from Mg–O stretching vibrations, is also important in terms of FTIR analysis of pure MgO. The spectrum is consistent with the data presented in the literature [21,22].

#### 2.1.2. Thermogravimetric Analysis (TGA)

**Figure 2:**
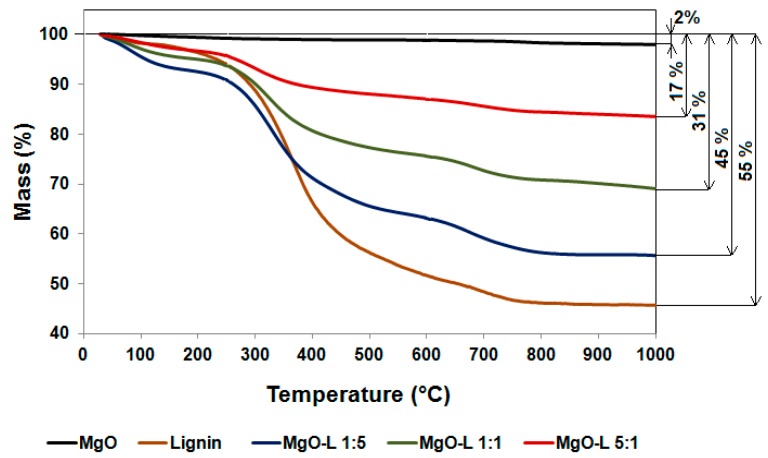
Thermogravimetric analysis (TGA) curves of pristine components (MgO and lignin) and inorganic–organic hybrid materials.

The assessment of thermal stability was based on the results of thermogravimetric analysis (TGA), which are presented in Figure 2. The very high thermal stability of pure MgO is noteworthy. The slight weight loss for the inorganic component was only caused by the desorption of water surface-bound to the oxide, mainly in the temperature range up to 200 °C. Very good stability of MgO is mainly associated with the method of its production. In the literature, many studies confirm that the thermal stability of MgO depends on the type and concentration of reagents used for its production, the methodology used for its preparation and the duration of the reaction [23,24]. The magnesium oxide used in this study definitely contributes to an improvement of the thermal stability obtained for all hybrid materials in each specified temperature range.

Detailed analysis of the results obtained allowed for the observation of three characteristic stages of decomposition of lignin under the influence of increased temperature, i.e., desorption of physically bound water (temperature up to ~210 °C), the disruption of aliphatic bonds in lignin with partial depolymerization of the compound (temperature of 210–600 °C) and final fragmentation of lignin, including the breakdown of the aromatic part of the biopolymer (temperature beyond 600 °C). The total weight loss for this compound was equal to 55% in the analyzed temperature range. Similar relationships have been obtained earlier, during the analysis of pure lignin [14,17,25,26]. Furthermore, based on the results obtained, it can be concluded that lignin causes a slight deterioration of the thermal stability of hybrid materials at temperatures beyond 300–350 °C, while at lower temperatures this stability is slightly improved. This is probably associated with the permanent effective connection of components through physical interactions, which has an additional, positive effect during polymer processing. As can be seen in Figure 2, the total weight loss for the analyzed hybrid materials was 17%, 31% and 45%, respectively, for systems with MgO–lignin ratios equal to 5:1 wt./wt., 1:1 wt./wt. and 1:5 wt./wt. On this basis, it was observed a significant influence of the composition of hybrid filler on the thermal stability of the final system. Similar observations can be found in the available literature [14,17,18,19]. 

#### 2.1.3. Dispersive-Morphological Properties

**Figure 3:**
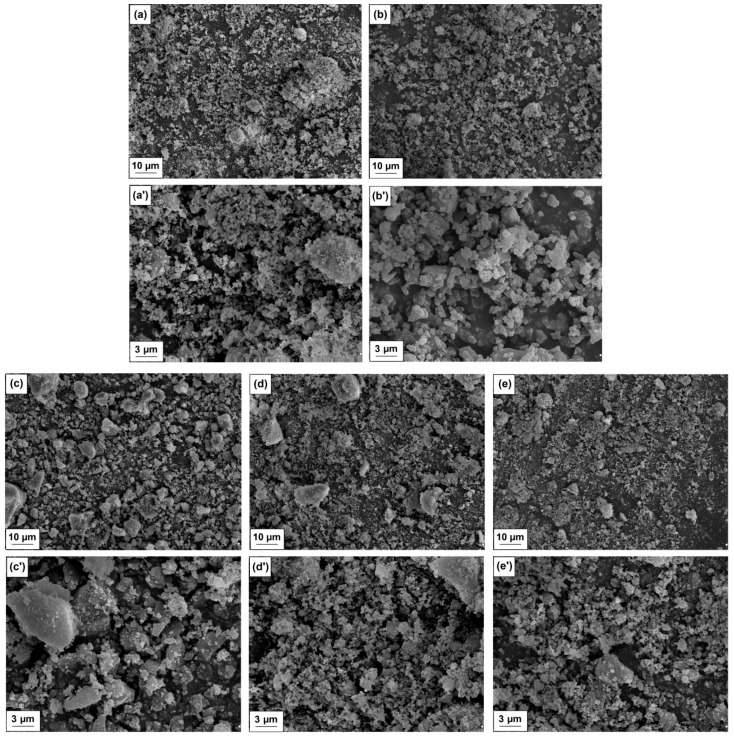
Scanning electron microscope (SEM) images of MgO (**a** and **a’**), lignin (**b** and **b’**), MgO-lignin hybrid materials with a weight ratio equal to 1:5 wt./wt. (**c** and **c’**), 1:1 wt./wt. (**d** and **d’**) and 5:1 wt./wt. (**e** and **e’**).

The morphological and microstructural characteristics of hybrid materials and pristine components were carried out using scanning electron microscopy (SEM). The SEM images obtained for pure magnesium oxide are presented in Figure 3a,a’, while the images obtained for lignin are presented in Figure 3b,b’. The images of hybrid materials with different ratios of components used (MgO-lignin equal to 5:1 wt./wt., 1:1 wt./wt. and 1:5 wt./wt.) obtained at two different magnifications are presented in Figure 3c–e,c’–e’.

Based on the analysis of SEM images, it can be observed that the lignin particles are larger than the magnesium oxide particles. Additionally, both products contain irregularly shaped particles. Furthermore, it can be concluded that the increase of the biopolymer ratio in the system causes a greater tendency to form aggregate and agglomerate structures, which impacts the final homogeneity of the samples (see Figure 3). The results obtained can be correlated with the data obtained using the Zetasizer Nano ZS apparatus, which was used to determine the particle size range. Based on the results obtained, it can be established that the particle size in case of MgO is in a relatively narrow range of 106–2670 nm, while for lignin this range is very wide and equal to 91–122 nm, 712–1110 nm and 2670–4800 nm. The addition of lignin to hybrid systems caused an increase of particle size, which was concluded based on the determined data presented in Table 1. The data obtained using the Mastersizer 2000 apparatus also confirms the above statement, as particles with a diameter below 1.2 µm account for 50% of the MgO sample volume, while 90% of the sample volume includes particles which do not exceed 2.1 µm. Pristine lignin was characterized by significantly larger particle diameters, in the case of which 10% of the particles possess a diameter below 2.1 µm, while particles with a diameter smaller than 5.2 µm and 8.3 µm account for 50% and 90% of the sample volume, respectively. In turn, according to the data presented in Table 1, the presence of lignin in hybrid materials caused an increase of the size of individual dispersion parameters.

Overall, the analysis carried out using three independent methods allowed us to estimate that the increase of the lignin ratio results in the presence of larger sizes of structures in the analyzed products. However, each of the materials is characterized by relatively good homogeneity, the microstructure of the systems consists of individual particles characterized by irregular shape, which exhibit the ability to form a relatively small amount of aggregates (<1 µm) and, consequently, secondary agglomerates (>1 µm).

#### 2.1.4. Porous Structure Parameters

Selected data regarding the parameters of the porous structure (Brunauer-Emmett-Teller BET surface area, average pore size and total pore volume) determined for MgO, lignin and the designed hybrid materials with different weight ratios of the used components are presented in Table 2. Based on the presented data, pristine magnesium oxide is characterized by the highest value of the BET surface area, in this case *A_BET_* = 99 m^2^/g. Lignin possesses a much smaller BET surface area—1 m^2^/g. In turn, individual hybrid systems assume values of this parameter in the range of 23–92 m^2^/g, and a tendency can be observed that the surface value decreases with higher lignin content in the system. A similar relationship can be observed for the total pore volume parameter, in case of which the results are in the range of 0.010–0.035 cm^3^/g. The average pore size, which is a significant parameter, did not differ significantly for the hybrid materials obtained (2.19–2.20 nm) and clearly depended on the presence of the inorganic component used for the preparation of hybrid materials. Similar BET surface area sizes have already been obtained in the literature before, and in case of pristine lignin were equal to ~1 m^2^/g [15,16,19]. In the case of the inorganic component, the values of this parameter strongly depend on the methodology of MgO synthesis, including the type and concentration of reagents, temperature and duration of the process [27,28].

It should be emphasized that the literature also includes reports which indicate that the ratio of individual components in the hybrid system has a very significant impact on the values of porous structure parameters obtained. Such systems include the widely studied SiO_2_–lignin systems [17,18,19] or the recently designed ZnO–lignin materials which were successfully used as fillers with potential antibacterial properties [14,29].

### 2.2. Characteristics of Polypropylene (PP)/MgO–Lignin Composites

#### 2.2.1. Wide-Angle X-ray Scattering (WAXS)

**Figure 4:**
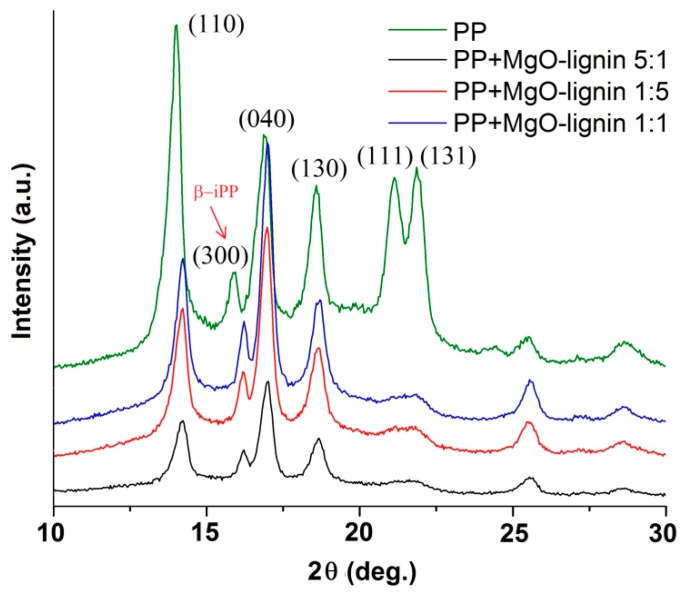
Wide-angle X-ray scattering (WAXS) patterns of composites.

Diffraction patterns presented in Figure 4 reveal peaks of both α and β forms of polypropylene. The peak at 2Θ = 16.2° is characteristic for β-PP while all the other peaks originate from α-PP [30]. It can be seen that the intensity of peaks, e.g., at 2Θ ≈ 14°, is not constant for different samples. When compared to an unfilled matrix, the composites were characterized by lower intensity of most of the peaks. This suggests that crystal planes were not formed as well in these materials as in the case of PP. Nonetheless, the amount of β form of PP in all composites was comparable (8–9%). In the literature [30], the α form of PP is defined as the most common, which occurs under conventional crystallization conditions, and the presence of the β form is attributed to specific processing conditions (presence of shearing forces) or addition of selective β-nucleating agents. In our research, such a β-nucleating agent was not used and the presence of β-PP is rather a result of shear forces present during the injection moulding process. Results presented in Table 3 confirm that addition of the MgO–lignin filler caused an increase of crystallinity degree (from 57% for PP to 53–58%). Composition of the filler did not significantly affect the formation of the crystalline phase of PP.

#### 2.2.2. Differential Scanning Calorimetry (DSC)

**Figure 5:**
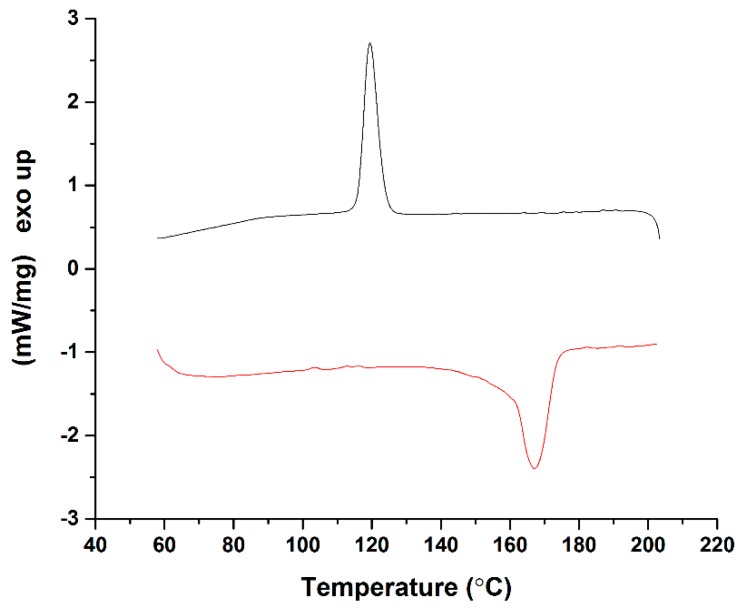
Differential scanning calorimetry (DSC) curves for polypropylene (PP) + MgO–lignin 1:5.

Differential scanning calorimetry (DSC) measurements were used to investigate the characteristic temperatures, crystallinity degree, and the kinetic parameters of polypropylene crystallization in composites. An exemplary thermogram is presented in Figure 5, while the data obtained from DSC curves are listed in Table 4.

Crystallization temperature of the tested composites ranged from 120 °C to 121 °C and melting temperature remained the same at 167 °C. These values are higher than for PP (T_m_ = 164 °C and T_c_ = 113 °C) and thus comparable with the literature data regarding polypropylene filled with lignocellulose fibers [31,32]. This confirms that the tested fillers acted as nucleating agents. DSC results also suggest that the content of MgO and lignin did not influence the crystallization and melting characteristics of composites. This observation is important in terms of processing of PP + MgO–lignin composites since it shows that such materials can be further used at typical processing parameters.

Based on the thermograms, the values of melting enthalpy and crystallinity degree were also determined. The calculated degree of crystallinity for composites was in the range of 37–41% and comparable to unfilled PP (see Table 4). The values of X_c_ obtained were similar, as in case of WAXS calculations. The composition of the filler used did not affect this parameter.

**Figure 6:**
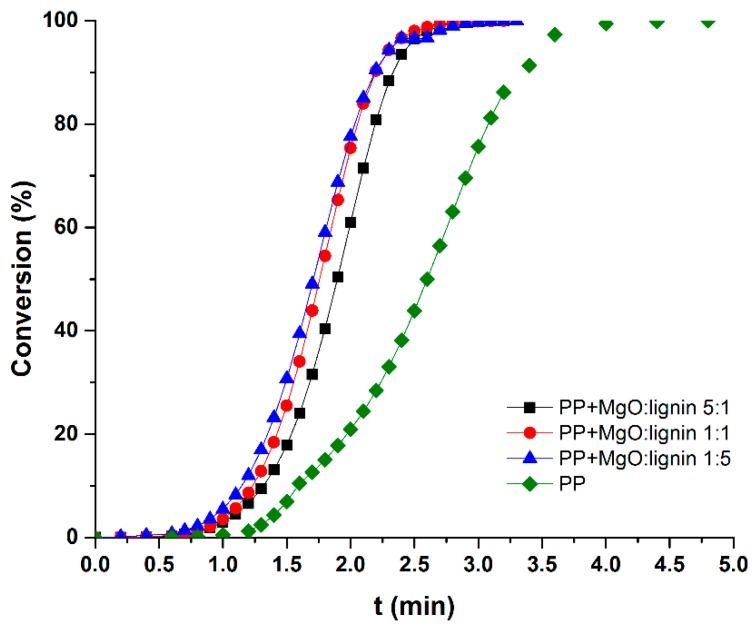
Crystal conversion rates for PP and composites.

The curves of the crystal conversion of PP and its composites are presented in Figure 6, and the calculated t_0.5_ are given in Table 5. Values of half-times of crystallization for composites do not show large discrepancies, ranging from 1.70 min to 1.90 min. In case of the reference sample, unfilled PP, this value was much higher −2.65 min. Taking into consideration all the aforementioned DSC results, including changes in Tc, it can be stated that all MgO–lignin fillers possess an ability to enhance the nucleating efficiency. On the contrary, in our previous studies regarding PP + ZnO–lignin composites [14], only samples filled with high lignin content (ZnO-lignin 1:5 wt./wt.) were characterized by lower values of t_0.5_ than PP. In this study, such behavior was observed for all composites. In order to further investigate these interesting results, polarized light microscopy (PLM) studies were performed.

#### 2.2.3. Polarized Light Microscopy (PLM)

**Figure 7:**
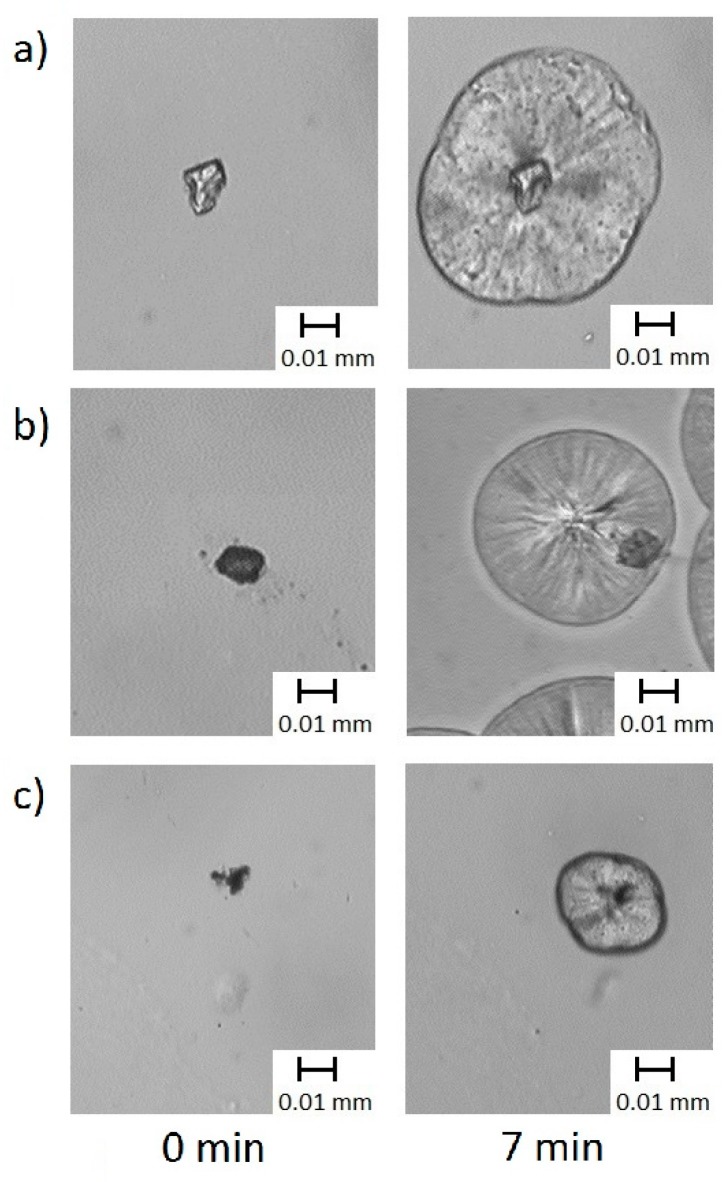
Crystallization of PP and its composites with (**a**) MgO–lignin 5:1 (**b**) MgO–lignin 1:5 (**c**) MgO–lignin 1:1 at 136 °C, at different times.

PLM studies were performed to assess the influence of the presence of MgO–lignin fillers on the crystallization of polypropylene. The PLM images of composites are shown in Figure 7.

It can be seen that spherulites were formed in case of all samples. However, the formation of transcrystalline structures was observed only in case of PP + MgO–lignin 1:1 wt./wt. and PP + MgO–lignin 5:1 wt./wt. In case of the composite with high lignin content, namely MgO–lignin 1:5 wt./wt. filler, spherulites tended to grow in the bulk of the polymer. Transcrystallization occurs when the polymer melt nucleates at crystalline surfaces. The presence of a transcrystalline layer (TCL) is known to influence the mechanical properties of composites [33,34]. This is a result of enhanced interfacial shear transfer between matrix and filler.

The induction time and growth rate of crystalline structures are presented in Table 6. Based on PLM photographs, it was calculated that the induction time for PP with MgO–lignin 5:1 wt./wt. and MgO–lignin 1:5 wt./wt. was the same, 0.5 min. Polymer filled with a MgO–lignin 1:1 wt./wt. hybrid exhibited remarkably higher induction time, 3.5 min. As visible on PLM photographs, TCL was formed in two out of three composites. In these samples the efficiency of the transcrystalline layer formation was diverse (1.4 µm/min and 5.4 µm/min). When nucleation occurred in the bulk of the polymer, in PP + MgO–lignin 1:5 system, the growth rate of spherulites was ca. 4.3 µm/min.

To sum up, materials with the highest MgO content exhibited high growth rate GR values. Filler with lower amount of metal oxide was associated with a certain reduction in GR and also increase of induction time IT. In the materials with the highest lignin content, the GR and IT values were similar to those obtained for the system with MgO-lignin 5:1 wt./wt., but no TCL formation was observed.

These results are consistent with our previous studies in which the influence of composition of hybrid filler on the nucleation ability of polymers (PP and PLA) was studied [14,17]. It was confirmed that composites with relatively high metal oxides content (ZnO, SiO_2_, MgO) are able to form TCL since crystallization occurs preferentially on fillers. On the other hand, the presence of high amounts of lignin favors the formation of spherulites, without TCL layer. Thus, it can be concluded that lignin itself had no significant effect on the morphology of PP.

#### 2.2.4. Mechanical Properties

The mechanical properties of PP and its composites are presented in Table 7. Young’s modulus of PP was equal to 1328 MPa and values of composites were slightly higher but still comparable (in the range from 1372 to 1382 MPa). Tensile strength (TS) of all the samples was approximated at 33 MPa. The most significant difference between the composites and PP was noted in terms of elongation at break. These results are similar to our results obtained for PP with ZnO–lignin filler [14] and correspond well with other reports [35]. Even though lignin is known to negatively impact the mechanical properties of composites [36,37], it was confirmed that in the case of our inorganic–organic fillers (both MgO–lignin and ZnO–lignin) this is not a major issue. The report of Zhou et al. [35] provides some information regarding the tensile properties of polypropylene filled with 0–5% of magnesium oxide. Young’s modulus and tensile strength of the tested composites did not change with the increase of the filler and was comparable with the unfilled polypropylene. It was also reported that PP–lignin composites exhibit higher YM than polypropylene but TS decreased as the filler content increased [38,39].

In this research, elongation at break was the only impaired parameter—it decreased from 91.8% for unfilled polypropylene to only 8% for composites, regardless of their composition. Zhou observed that the value of EB increased in systems containing up to 1% filler and then drops [35]. The presence of stiff particles of the filler can lower the elasticity of the material due to too strong filler–polymer interactions [40]. In consequence, propagation breaks and composites became weaker. Results of tensile tests presented in this research correspond well with aforementioned reports. However, our findings are promising because even though MgO-lignin filler was used, Young’s modulus and tensile strength of the composites produced remained comparable or even slightly higher than in case of unfilled polypropylene.

## 3. Materials and Methods

### 3.1. Materials

Magnesium oxide (Sigma Aldrich, Steinheim am Albuch, Germany) and kraft lignin (Sigma-Aldrich, Germany) were used to obtain the hybrid materials. The final products in the form of MgO–lignin hybrids were produced at three different ratios: (i) 1 part by weight of MgO per 5 parts by weight of lignin (1:5), (ii) 1 part by weight of MgO per 1 part by weight of lignin (1:1) and (iii) 5 parts by weight MgO per 1 part by weight of lignin (5:1).

Polypropylene (isotactic polypropylene, Moplen, BasellOrlen Polyolefines Sp. zo.o., Płock, Poland) was used to produce composites.

### 3.2. Preparation of MgO–Lignin Hybrid Materials

The inorganic and organic components were combined using a mechanical method, which has already been used in our previous works [14,17,18,19]. After placing the appropriate amounts of magnesium oxide and biopolymer in a planetary ball mill, the process of grinding the ingredients began. In order to obtain a homogeneous mixture, the process was planned for a period of 2 h. Within a given time, cyclic breaks were carried out every 30 min, for a period of 5 min, to prevent uncontrolled thermal degradation of the organic part of the hybrid. Before performing the full physicochemical and dispersive-morphological characteristics, the final hybrid materials and the components used were sieved using a 40 µm sieve to ensure full homogeneity of the systems.

### 3.3. Characterization of Inorganic–Organic Hybrid Materials

Identification of bands corresponding to the characteristic groups of the analyzed products was carried out using Fourier transform infrared spectroscopy (FTIR). This technique enables the registration of vibration bands of functional groups of organic and inorganic compounds in the wavenumber range of 4000–400 cm**^−^**^1^. FTIR spectra were recorded using a Vertex 70 spectrophotometer (Bruker Optics GmbH, Ettlingen Germany). The camera provides a very high resolution of sample scanning, which exceeds 0.5 cm**^−^**^1^. The use of such a measuring system enabled quick and selective scanning of prepared samples in the form of KBr pellets. Approximately 250 mg of anhydrous potassium bromide was used to make the pellets, which was triturated with 2 mg of the test sample. The prepared powder was transferred to a hydraulic press (Specac Ltd., Orpington, UK) and pressed at 10 MPa for 10 min. As a result, a crystalline pellet was obtained, which was placed in a spectrophotometer measuring cuvette and examined.

The assessment of the thermal stability of the samples was carried out by using thermogravimetric analysis (TGA). The weight loss of samples in a given temperature range was determined using a Jupiter STA 449 F3 analyzer (Netzsch GmbH, Selb, Germany). The evaluation was carried out in the temperature range from 20 to 1000 °C, with steps of 10 °C/min, under nitrogen flow (10 mL/min). In order to conduct the TGA analyzes, approximately 5 mg of test sample was used. Alumina was used as the reference substance, which does not undergo thermal changes under the test conditions. The TGA analysis is based on recording of mass changes of the tested material under the influence of controlled heating to a given temperature, which is graphically presented in the form of a thermogram.

The EVO40 scanning electron microscope (SEM) from Zeiss AG, Jena, Germany was used to assess the morphology and microstructure of pristine components and hybrid systems. The apparatus accelerates the excited electrons in the voltage range from 0.2 to 30 kV. As a result, it offers good resolution of the scanned image of the sample surface. Prior to taking a picture of the surface of the material, the sample was covered with gold using a Balzers PV205P apparatus (Oerlikon Bazers Coating SA, Biel Switzerland). Additionally, the particle size ranges and dispersion parameters were determined using two devices: Zetasizer Nano ZS and Mastersizer 2000 (both Malvern Instruments Ltd., Malvern, UK). These devices allow for the measurement of particle sizes in the range of 0.6–6000 nm (non-invasive back light scattering method (NIBS)) and 0.2–2000 µm (laser diffraction).

The porous structure parameters, such as: BET surface area (*A_BET_*), total pore volume (*V_p_*) and average pore size (*S_p_*) were also determined in the framework of the study. The measurements were carried out using the ASAP 2020 apparatus, Micromeritics Instrument Co., Norcross, GA, USA. The BET surface area was determined using the Brunauer-Emmett-Teller method, while the total volume and average pore size were determined according to the BJH (Barret–Joyner–Halenda) algorithm. The measurement started with degassing of the sample in a vacuum chamber at 120 °C for 4 h. Next, purified nitrogen was introduced, which filled the capillaries of the material, and was then condensed at −196 °C.

### 3.4. Preparation of Polypropylene Composites

Polypropylene and 3% (wt./wt.) of a hybrid filler were melt-blended in a single screw extruder (McNeil REPIQUET, model Fairex, Aulnay-sous-Bois, France) and then cut with a knife mill (25-16/TC-SL, TRIA, Novi, MI, USA). Injection molding machine (ENGEL type HLS 20/80, ø = 22 mm) was used to produce specimens for further characterization. The extrusion process was performed at a barrel temperature of 140–210 °C and a screw rotation speed of 120 rpm. Melt flow index (at 2.16 kg, 230 °C) for composite pellets was (g/10 min): 2.7, 4.9, 4.5, and 4.0 for PP, PP + MgO–lignin 5:1, PP + MgO–lignin 1:1, PP + MgO–lignin 1:5, respectively. The injection molding process was carried out in temperature of 180–210 °C, mold temperature was 60 °C. Cooling time was set to 30 s.

### 3.5. Characterization of Polypropylene Composites

The supermolecular structure of composite materials was analyzed by means of wide-angle X-ray scattering (WAXS) using CuKα radiation at 30 kV and 20 mA anode excitation. The X-ray diffraction patterns were recorded for the angle range from 10 to 30° with the step of 0.04°/3 s.

Thermal properties of materials in the form of films were evaluated using differential scanning calorimetry (DSC 1, Mettler Toledo, Columbus, OH, USA) under nitrogen atmosphere. For non-isothermal crystallization investigations, the samples were first heated from 40 to 210 °C at the rate 20 °C/min and kept at this temperature for 3 min to eliminate the previous thermal and/or mechanical history. Then, the samples were quenched to 40 °C at the rate 5 °C/min and kept at 40 °C for 1 min. This procedure was repeated two times and the second run was used in the calculations. Based on the determined values for the enthalpy of crystallization (H), the extent of crystallization (crystal conversion), α was calculated (Equation (1)):

(1) $$\alpha =\frac{\int_{0}^{t}\left(\frac{dH}{dt}\right)\times dt}{\int_{0}^{1}\left(\frac{dH}{dt}\right)\times dt}$$

Based on the curves of α = f(t), the half-time of crystallisation (t_0.5_) was determined as the time when crystal conversion was equal to 50%. The crystallinity degree (X_c_) of materials was evaluated according to Equation (2).

(2) $${\;X}_{c}=\left(\frac{\Delta \mathrm{Hm}}{\Delta \mathrm{Hm}{}^{\circ}\times \left(1-\frac{\%\mathrm{wt}\;\mathrm{filler}}{100}\right)}\right)\times 100$$

where: ΔH_m_ is the melting enthalpy (from second heating scan), ΔH_m_° is the melting enthalpy of a 100% crystalline polymer matrix (209.0 J/g for PP [41]) and %wt. filler is the filler weight percentage. Furthermore, characteristic temperatures such as melting (T_m_) and crystallization (T_c_) temperatures were defined.

The isothermal crystallization of PP in the presence of lignin, MgO, and hybrid filler was carried out using the hot-stage optical (Linkam TP93, Linkam Scientific Instruments Ltd, Epsom, UK) and a polarizing optical microscope Nikon Eclipse (LV100POL, USA) equipped with a Panasonic CCD camera (GP-KR222, Newark, NJ, USA). The samples were first heated to 200 °C at the rate 30 °C/min and kept at this temperature for 3 min to eliminate the previous thermal and/or mechanical history. Then, the samples were cooled at 10 °C/min to 136 °C at which isothermal crystallization of the PP was possible. Dry nitrogen was introduced to eliminate any possible degradation during measurement. Photographs were taken every 10 s. Analysis of the photographs was conducted by means of a MultiScan program and the density of nuclei of PP and its composites was determined as a result.

Tensile properties of composites were defined using a Zwick and Roell Allround-Line Z020 TEW testing machine (Ulm, Germany). The tests were performed in accordance with ISO 527 1-2, the test speed was equal to 50 mm/min. In each case, the arithmetic mean of at least seven replicate determinations was taken into consideration.

## 4. Conclusions

Functional MgO–lignin hybrid materials were designed and prepared using the method of mechanical grinding of components. For this purpose, a planetary ball mill was used to ensure that the final products were characterized by adequate homogeneity. Based on the FTIR spectroscopy studies, it was concluded that there was a stable physical interaction in the form of hydrogen bonds between MgO and lignin. This is confirmed by changes (shifts) in the absorption maxima of individual bands of characteristic groups, primarily including hydroxyl groups. On this basis, it can be concluded that the hybrid materials belonging to class I were successfully obtained. Additionally, the morphological, microstructural and dispersion characteristics allowed us to confirm that the hybrid systems are characterized by good homogeneity, with a small tendency to create larger aggregate and agglomerate structures that do not affect the final homogeneous distribution of the filler in the polymer composite. The analysis of thermal stability allowed us to conclude that hybrid systems are characterized by a relatively small mass loss in a wide temperature range, which is lower with the increase of the inorganic component content in the system. The nature of the porous structure depended on the ratio of the components used and the BET surface area, while the total pore volume increased with the increase of the inorganic component ratio.

Polypropylene and its composites with MgO–lignin hybrid fillers were analyzed by polarized light microscopy. The results obtained indicate that the hybrid filler, in which magnesium oxide is the dominant component, promotes the growth of the transcrystalline layer. Polymer composites were also subjected to thermal analysis and wide-angle X-ray diffraction scattering. The results confirmed that melting and crystallization temperatures as well as degree of crystallinity of all composites were comparable. However, in comparison to unfilled PP, the melting and crystallization temperatures of composites were higher. Both polymorphic forms of isotactic polypropylene (α-iPP and β-iPP) were found in all composites. The percentage of the β form remained unchanged. The performed tensile tests showed no effect of the composition of hybrid fillers on the value of Young’s modulus and tensile strength.

## Figures and Tables

**Table 1:** Dispersive properties of MgO, lignin and inorganic-organic hybrid materials.

Sample Name			Dispersive Properties			
	Particle Size Distribution from Zetasizer Nano ZS (nm)	Polydispersity Index (PDI) from Zetasizer Nano ZS	Particle Diameter from Mastersizer 2000 (μm)			
			d(0.1) ^1^	d(0.5) ^2^	d(0.9) ^3^	D [4.3] ^4^
MgO	106–2670	0.286	0.5	1.2	2.1	1.4
Lignin	91–122, 712–1110, 2670–4800	0.691	2.1	5.2	8.3	6.5
MgO–lignin (1:5 wt./wt.)	122–615, 1720–5560	0.587	1.6	3.8	4.5	4.0
MgO–lignin (1:1 wt./wt.)	106–615, 1484–4800	0.522	1.4	3.1	4.2	3.2
MgO–lignin (5:1 wt./wt.)	106–712, 1110–2670	0.461	1.4	2.9	4.1	3.0

^1^ d(0.1)—10% of the volume distribution is below this value diameter ^2^ d(0.5)—50% of the volume distribution is below this value diameter ^3^ d(0.9)—90% of the volume distribution is below this value diameter ^4^ D [4.3]—average particle size in examined system.

**Table 2:** Dispersive properties of MgO, lignin, and inorganic–organic hybrid materials.

Sample Name	Porous Structure Properties		
	*A_BET_* (m^2^/g) ^1^	*S_p_* (nm) ^2^	*V_p_* (cm^3^/g) ^3^
MgO	99	2.20	0.037
Lignin	1	9.21	0.001
MgO–lignin (1:5 wt./wt.)	23	2.19	0.010
MgO–lignin (1:1 wt./wt.)	54	2.19	0.021
MgO–lignin (5:1 wt./wt.)	92	2.20	0.035

^1^***A_BET_***—Brunauer-Emmett-Teller surface area ^2^***S_p_***—mean size of pores ^3^***V_p_***—total volume of pores.

**Table 3:** Crystallinity degree of composite materials.

Sample Name	*X_c_* (%) ^1^
PP	
PP + MgO-lignin 5:1	58
PP + MgO-lignin 1:5	57
PP + MgO-lignin 1:1	53

^1^***X_c_***—crystallinity degree.

**Table 4:** Data obtained from DSC curves.

Sample Name	*ΔH_m_* (J/g) ^1^	*T_m_* (°C) ^2^	*T_c_* (°C) ^3^	*X_c_* (%) ^4^
PP	76	164	113	36
PP + MgO–lignin 5:1	78	167	120	37
PP + MgO–lignin 1:5	82	167	121	39
PP + MgO–lignin 1:1	86	167	120	41

^1^***ΔH_m_***—melting enthalpy ^2^***T_m_***—melting temperature ^3^***T_c_***—crystallization temperature ^4^***X_c_***—crystallinity degree

**Table 5:** Crystallization half-times of PP and its composites.

Sample Name	*t_0.5_* (min) ^1^
PP	2.65
PP + MgO-lignin 5:1	1.90
PP + MgO-lignin 1:5	1.70
PP + MgO-lignin 1:1	1.77

^1^***t_0.5_***—crystallization half-time.

**Table 6:** Growth rate and induction times of PP and its composites calculated from polarized light microscopy (PLM).

Sample Name	Growth Rate of Transcrystalline Layer (or Spherulite *) (µm/min)	Induction Time (min)
PP + MgO–lignin 5:1	5.4	0.5
PP + MgO–lignin 1:5	4.3*	0.5
PP + MgO–lignin 1:1	1.4	3.5

* presence of spherulites.

**Table 7:** Mechanical properties of PP and its composites.

	YM ^1^ [MPa]	TS ^2^ [MPa]	EB ^3^ [%]
PP	1328 ± 22.4	33.8 ± 0.40	91.8 ± 4.4
PP + MgO-lignin 5:1	1382 ± 15.1	32.8 ± 0.31	8.0 ± 0.4
PP + MgO-lignin 1:5	1375 ± 21.8	33.2 ± 0.20	8.9 ± 0.1
PP + MgO-lignin 1:1	1372 ± 4.4	33.0 ± 0.17	8.2 ± 0.3

^1^ YM—Young’s modulus ^2^ TS—tensile strength ^3^ EB—elongation at break.
